# Beyond Borders: Investigating the Impact of COVID-19 Anxiety and Eating Attitudes on Psychological Well-Being and Physical Activity Objectives in Poland and China

**DOI:** 10.3390/nu17010041

**Published:** 2024-12-26

**Authors:** Jianye Li, Dominika Maria Wilczyńska, Małgorzata Lipowska, Ariadna Beata Łada-Maśko, Bartosz M. Radtke, Urszula Sajewicz-Radtke, Bernadetta Izydorczyk, Taofeng Liu, Zitong Wang, Junyu Lu, Mariusz Lipowski

**Affiliations:** 1 Faculty of Physical Education, Gdańsk University of Physical Education and Sport, Górskiego 1, 80-336 Gdańsk, Poland; jianye.li@awf.gda.pl (J.L.); zitong.wang@awf.gda.pl (Z.W.); junyu.lu@awf.gda.pl (J.L.); 2 Faculty of Social and Humanities, WSB Merito University Gdansk, 80-266 Gdańsk, Poland; dominika.wilczynska@gdansk.merito.pl; 3 Institute of Psychology, University of Gdańsk, 80-309 Gdańsk, Poland; malgorzata.lipowska@ug.edu.pl (M.L.); ariadna.lada@ug.edu.pl (A.B.Ł.-M.); 4 Laboratory of Psychological and Educational Tests, 80-239 Gdańsk, Poland; radtke@pracowniatestow.pl (B.M.R.); sajewicz-radtke@pracowniatestow.pl (U.S.-R.); 5 Institute of Psychology, Jagiellonian University, 30-060 Krakow, Poland; bernadetta.izydorczyk@uj.edu.pl; 6 School of Physical Education, Zhengzhou University, Zhengzhou 450001, China; liutaofeng@zzu.edu.cn

**Keywords:** COVID-19, well-being, eating attitudes, physical activity objectives

## Abstract

Background/Objectives: The mechanisms linking eating attitudes to well-being and physical activity objectives have increasingly attracted the attention of researchers in recent years. This research is particularly significant in the context of the COVID-19 pandemic, which has profoundly disrupted eating habits, exercise routines, and psychosocial well-being across the globe. Additionally, these variables are influenced by cultural dimensions, such as individualism in Poland and collectivism in China. These two countries represent distinct approaches to social health and well-being during the pandemic, offering valuable comparative insights into how cultural contexts shape mental and physical health behaviors; Methods: The study included 644 Polish and 690 Chinese participants. It utilized the Psychological Well-Being Scale (PWBS), the Coronavirus Anxiety Scale (CAS), the Eating Attitudes Test (EAT-26), and the Inventory of Physical Activity Objectives (IPAO); Results: The results indicate that both COVID-19 anxiety and eating attitudes fully mediate the relationship between well-being and physical activity objectives. Full mediation implies that the observed relationship between well-being and physical activity objectives operates entirely through the mediators. Notably, the mediating effect of COVID-19 anxiety was observed only in the Chinese sample, highlighting cultural differences in coping mechanisms and societal responses to anxiety. Cultural differences significantly influenced well-being and physical activity objectives, while eating disorders remained unaffected by cultural and social differences. Additionally, a significant positive correlation was found between COVID-19 anxiety, eating attitudes, and health-related physical activity objectives, underscoring the interplay between mental health and physical activity; Conclusions: These findings underscore the importance of addressing anxiety and eating attitudes to enhance well-being and physical activity behaviors. The study provides a strong theoretical basis for targeted interventions tailored to cultural contexts. Potential limitations include the reliance on self-reported data and differences in demographic characteristics between the Polish and Chinese samples, which may affect generalizability.

## 1. Introduction

Since the first confirmed case of COVID-19 was found in Wuhan, China, in 2019, it has undergone widespread transmission and spread around the world, seriously affecting the daily lives of people around the world. Although positive responses and lockdown measures implemented by various countries have helped slow the spread of the virus and played an effective role in protecting public health, these prolonged lockdowns have also led to widespread anxiety and negative psychological and physical effects [1,2,3]. Several studies have shown the psychological effects of COVID-19, mainly in the form of varying degrees of anxiety, depression, insomnia, eating disorders, increased stress, reduced well-being, and other negative psychological conditions across different groups. The research also indicated that women and young people had higher rates of negative psychological states [4,5].

Some scholars have suggested that COVID-19 anxiety contains two main dimensions such as social interaction fear and illness anxiety. Both show a significant negative correlation with mental health and general physical health [6,7]. Further research has highlighted that negative psychological states have a significant correlation with physical health, i.e., the more severe the negative emotions, the more likely they are to cause disruptions in daily behaviors. Studies have indicated that because people’s behavioral activities are restricted during COVID-19 and they have to engage in prolonged home isolation measures, people will mitigate the wide range of negative emotions caused by COVID-19 anxiety by increasing the frequency of behaviors such as smoking, drinking alcohol, and overeating [8]. In studies of COVID-19 anxiety and psychological well-being, several researches have pointed to a significant increase in the prevalence of mental illness during the COVID-19 epidemic [9,10,11].

The review of previous studies demonstrates that COVID-19 anxiety can negatively impact people’s psychological well-being (PWB), and physical health, highlighting the importance of maintaining good psychological well-being. Two researchers also point out that psychological well-being requires perceived involvement in life’s challenges [12,13]. In a cross-sectional study in Japan, researchers concluded that the PWB scale was a good indicator of young people’s well-being, while noting that the main causes of negative emotions such as anxiety and depression are due to a lack of well-being [14]. In the COVID-19 study on anxiety and well-being some scholars have linked psychological well-being and satisfaction to the moderating effects of anxiety and mental health [15]. There are simultaneously studies that suggest that eating disorders are not only detrimental to an individual’s physical health, but also have a serious impact on overall levels of psychological well-being [16]. In this context, it becomes crucial to pay special attention to the relationship between an individual’s mental health and physical health. Understanding how to maintain good eating habits and physical health in anxious and stressful environments is crucial.

Physical activity is an acknowledged better modality among the moderating measures of psychological well-being in the current study. Exercising at home during COVID-19 can reduce sedentary time as well as the risk of illness, and have a positive effect on psychological well-being [17]. A study on Baduanjin exercise has shown that it promotes physical and psychological well-being, reduces perceived anxiety about COVID-19 and reduces the risk of back pain during the blockade, thereby improving psychological well-being [18]. However, some studies have also shown that COVID-19 anxiety can alter the content and objectives of people’s physical activity. This is mainly evident in the decrease in physical activity levels, which is more significant in men. Secondly, age, gender, culture, and education are also affected to some extent by COVID-19 anxiety, thus changing the pattern and content of physical activity [19,20]. According to several studies it can be assumed that there is a moderating effect and correlation between COVID-19 anxiety and mental and physical health and physical activity. The maintenance of physical activity levels is also one of the crucial goals for many people [21]. However, many people find their exercise habits challenged due to the constraints of the epidemic and concerns about public spaces. Therefore, it is of great theoretical and practical importance to examine how feasible and realistic physical activity goals can be set in this context, and how these goals interact with psychological well-being.

Research indicates that anxiety can disrupt healthy eating patterns and reduce physical activity levels, thereby affecting overall well-being. For instance, studies have shown that anxiety during the pandemic has led to significant changes in eating behaviors, such as increased emotional eating and unhealthy food choices, which in turn affect physical health and activity levels [22]. Therefore, Based on these findings, it is hypothesized that COVID-19 anxiety and eating attitudes serve as mediators in the relationship between well-being and physical activity objectives.

Cultural differences play a crucial role in shaping psychological constructs and behaviors. Hofstede’s cultural dimensions theory provides a framework for understanding how cultural contexts influence values, behaviors, and attitudes. For example, individualistic cultures (like Poland) may emphasize personal autonomy and self-expression, while collectivistic cultures (like China) may prioritize social harmony and community. These cultural orientations can influence responses to stress and anxiety [23]. In individualistic cultures like Poland, where personal freedom and autonomy are highly valued, lockdown measures might have heightened anxiety due to perceived restrictions on personal rights. Conversely, in collectivistic cultures like China, where community welfare often takes precedence, compliance with public health measures might mitigate anxiety, as individuals perceive their actions as part of a collective effort.

In individualistic cultures, stress and anxiety might lead to emotional eating or irregular eating patterns, as people prioritize self-comfort during crises. In contrast, collectivistic cultures may exhibit a preference for family meals or traditional dietary practices that provide a sense of normalcy and community, potentially buffering negative changes in eating attitudes.

Individualistic cultures may emphasize personal fitness goals, which might decline during lockdowns due to restricted access to gyms or outdoor spaces. Collectivistic cultures, however, might maintain activity levels through community-driven or family-oriented activities, as the focus remains on collective psychological well-being.

Additionally, cultural norms around body image, diet, and physical activity differ significantly, impacting eating attitudes and physical activity levels [24]. Therefore, a reasonable second hypothesis is that significant differences in psychological well-being, COVID-19 anxiety, eating attitudes, and physical activity objective scores are expected between Polish and Chinese participants due to these cultural influences.

The biopsychosocial model of health posits that biological, psychological, and social factors interact to influence overall health and well-being [25]. This model suggests that psychological states such as anxiety can impact physical health behaviors, including eating habits and physical activity. The biopsychosocial model emphasizes the role of psychological factors in health, which aligns with self-determination theory (SDT), suggesting that fulfillment of basic psychological needs enhances motivation for healthy behaviors. The self-determination theory (SDT) also supports this hypothesis, proposing that fulfilling basic psychological needs (autonomy, competence, and relatedness) enhances motivation for health-promoting behaviors [26]. Empirical evidence indicates that higher levels of well-being are associated with better physical health outcomes and healthier behaviors [27]. Thus, significant correlations between well-being, COVID-19 anxiety, eating attitudes, and physical activity objectives are third hypothesis based on these theoretical frameworks.

Current research on COVID-19 anxiety, eating disorders, and physical and mental health has been conducted in independent countries or single-sample studies, with relatively little cross-cultural research between multiple countries. This study explores the impact of psychological well-being on physical activity objectives and examines whether COVID-19 anxiety and eating attitudes have a mediating effect.

## 2. Materials and Methods

### 2.1. Research Design

A quantitative, correlational, and descriptive cross-sectional study was conducted as part of a large international research project, registered in the Protocol Registration (ClinicalTrials.gov; http://clinicaltrials.gov/ct2/show/NCT04432038, accessed on 1 April 2020). The research team initiated the study by providing ethics training and detailed research procedures to researchers with expertise in public health and survey methodologies. Participants meeting the inclusion criteria received an informed consent form and an online link to the questionnaire. To expand the sample size, participants were encouraged to invite friends and family who met the inclusion criteria, utilizing a snowball sampling technique. All participants gave informed consent before completing the questionnaires. The questionnaire was translated into Polish and Chinese, and its reliability and validity were confirmed using the KMO and Bartlett’s tests.

### 2.2. Participants and Procedure

The present study involved 1334 adult citizens from China and Poland, comprising 803 males and 531 females, all of whom were free from chronic illnesses or physical disabilities. The average age of the participants was M = 28.89 years, with an age range of 18 to 74 years (standard deviation [SD] = 10.01 years). Participants were recruited using convenience sampling, which involves selecting readily available individuals, and purposive sampling, which targets individuals meeting specific criteria. The inclusion criteria were as follows: participants had to be aged over 18 years, hold Chinese or Polish nationality, have no physical disabilities or medical conditions impeding physical activity, and have no history of treatment for eating disorders or COVID-19-related anxiety. A questionnaire verified these criteria to ensure proper exclusion of ineligible individuals.

A power analysis for an F-test was conducted using G*Power software (version 3.1), indicating that a minimum sample size of 511 would be required to achieve a statistical power of at least 0.9, with an alpha level of 0.05 and a medium effect size (d = 0.5). All questionnaires used in the study were administered electronically, with data collection occurring between April and August 2020, coinciding with the global outbreak of the COVID-19 pandemic. The questionnaires were distributed online via electronic survey platforms and disseminated through student samples from the academic institutions affiliated with the research team.

The survey included participants’ basic demographic information and responses to four psychological scales: the Psychological Well-Being Scale, the COVID-19 Anxiety Scale, the Eating Attitudes Test, and the Physical Activity Objectives scale. The estimated time for questionnaire completion was approximately 20 min.

The study protocol was approved by the Research Ethics Committee of the Institute of Psychology at the University of Gdańsk, Poland (decision number: 33/2020), and was registered in the ClinicalTrials.gov Protocol Registration and Results System; https://clinicaltrials.gov/ct2/show/NCT04432038 (accessed on 1 April 2020).

### 2.3. Instruments

This study employed four validated and well-established measurement scales, namely, the PWBS, CAS, Eat-26, and IPAO. The study team adhered to the Guidelines for Cross-Cultural Adaptation of Self-Report Measures to ensure the linguistic and cultural validity of the scales [28] to translate all scales into Polish and Chinese. The translation process was conducted under rigorous conditions, involving thorough discussions and comparisons among the research team members to ensure the accuracy and cultural appropriateness of the translated content.

#### 2.3.1. Inventory of Physical Activity Objectives (IPAO)

The IPAO, designed by Lipowski [29], assesses physical activity objectives across three dimensions: Health, Wellbeing, and Stress Reduction. It consists of 12 items, scored on a 5-point Likert scale (1 = completely disagree, 5 = completely agree), with higher scores indicating greater engagement in the respective objectives. In this study, the internal consistency (Cronbach’s alpha) was 0.90 for the Polish sample and 0.88 for the Chinese sample.

#### 2.3.2. The Psychological Well-Being Scale (PWBS)

The PWBS, developed by Ryff [30], measures psychological well-being across six dimensions: self-acceptance, environmental mastery, positive relations, purpose in life, personal growth, and autonomy. Each dimension includes three items, rated on a 6-point Likert scale (1 = strongly disagree, 6 = strongly agree), with higher scores reflecting stronger well-being. Due to low loading factors (<0.5) for personal growth and autonomy in the Polish sample, these items were excluded. The Cronbach’s alpha was 0.90 for the Polish sample and 0.88 for the Chinese sample.

#### 2.3.3. Coronavirus Anxiety Scale (CAS)

The CAS, introduced by Lee [31], evaluates anxiety levels related to the COVID-19 pandemic through five items addressing physiological symptoms of fear and anxiety. Responses are scored on a 5-point Likert scale (1 = not at all, 5 = nearly every day over the past two weeks), with higher scores indicating greater anxiety. The Cronbach’s alpha was 0.92 for the Chinese sample and 0.90 for the Polish sample, reflecting strong reliability.

#### 2.3.4. Eating Attitudes Test-26 Items (EAT-26)

The EAT-26, a widely used self-report instrument [32], assesses disordered eating behaviors, weight concerns, and body image issues through 26 items. Participants rate their responses on a 6-point Likert scale (1 = never, 6 = once a day or more), with higher scores indicating greater disordered eating tendencies. In this study, Cronbach’s alpha was 0.91 for the Polish sample and 0.88 for the Chinese sample, demonstrating high reliability.

### 2.4. Data Analysis

The data from the questionnaire were analyzed and validated using SPSS 26.0 (IBM, Armonk, New York, NY, USA) and AMOS 26.0 (IBM, Armonk, New York, NY, USA). Data visualization plots were generated in JupyterLab using Matplotlib (https://jupyter.org/try-jupyter/lab/, accessed on 26 November 2024). The analysis was conducted in the following stages:

Stage 1: Descriptive statistics were obtained by measuring the mean, standard deviation, and effect sizes of variables in the research model. Differences in wellbeing, COVID-19 anxiety, eating attitude, and physical activity objectives across cultures were elucidated, and effect values were calculated.

Stage 2: A loading factor analysis was conducted on the dimensions of all variables. Items with loading factors below 0.5 were excluded, which ensured that only items with sufficient explanatory power contributed to the overall analysis, thereby improving the reliability and validity of the measurement model.

Stage 3: In constructing the research model, AMOS 26.0 was used. The PWBS was considered the independent variable, IPAO the dependent variable, and CAS and EAT-26 as mediator variables. This stage aimed to investigate the influence pathways and the mediating roles of CAS and EAT-26 in the model, with a comparison of mediating effects between the two countries.

Stage 4: Multiple regression analyses were conducted to explore the relationship between predictor variables and IPAO, using demographic characteristics and PWBS as predictor variables.

Stage 5: ANOVA was performed to assess significant differences in the scores of the two countries across all scales.

Stage 6: Pearson’s correlation was used to measure the strength of correlation between all scales, and differences between the two countries were elaborated.

## 3. Results

### 3.1. Descriptive Analysis

Table 1 illustrates the descriptive analysis of all variables. Indicators with large effect sizes (Cohen’s d ≥ 0.80) [33] between Poland and China were observed in the following: Age: A large effect size (d = 0.82) indicates significant differences in the average age of participants between the two countries. Well-being: The dimension of Autonomy showed a large effect size (d = 0.68), reflecting notable differences in this aspect of psychological well-being; COVID-19 Anxiety (CAS): Large effect sizes were found in the following items: I felt dizzy, lightheaded, or faint when I read or listened to news about COVID-19 (d = 0.84); had trouble falling or staying asleep because I was thinking about COVID-19 (d = 0.91). I lost interest in eating when I thought about or was exposed to information about COVID-19 (d = 0.93); Eating Attitudes (EAT-26): The Dieting subscale had a large effect size (d = 0.81); Physical Activity Objectives (IPAO): The Health subscale showed a moderate-to-large effect size (d = 0.63).

This detailed breakdown highlights the specific variables contributing to significant differences between Polish and Chinese participants. Table 1 provides the corresponding means, standard deviations, and effect sizes for further reference.

### 3.2. Measurement Model Analysis

This study employed a numerical criterion of 0.50 as the acceptable threshold for factor loadings (see Table 2). Any indicators with factor loadings below this threshold were excluded from further analysis to ensure the robustness of the measurement model. Specifically, the indicators “Purpose in life” and “Personal growth” were excluded because their factor loadings fell below the 0.50 threshold. This statistical criterion ensures that only factors with sufficient explanatory power are retained, thereby enhancing the reliability and validity of the measurement instrument.

From a theoretical perspective, retaining only those indicators with higher factor loadings strengthens the construct validity of the model. The exclusion of these two factors minimizes potential noise in the data and ensures that the final model accurately reflects the key dimensions of psychological well-being. Consequently, “Purpose in life” and “Personal growth” will not be included in subsequent analyses.

### 3.3. Structural Model Analysis

**Figure 1:**
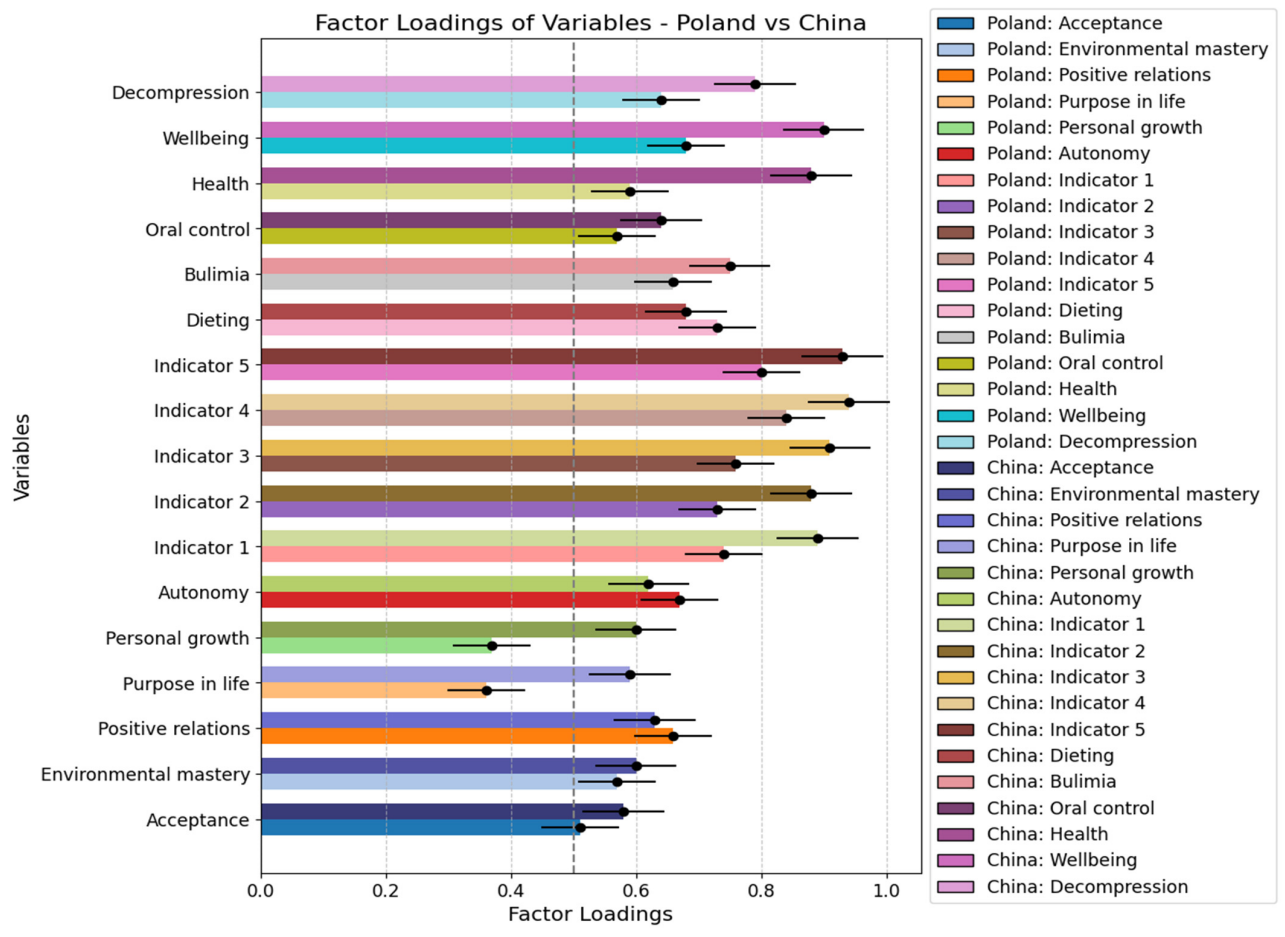
Factor Loadings of Variables in Poland and China. Values represent the factor ladings.

The model was constructed with PWBS as the independent variable, IPAO as the dependent variable, and CAS and EAT-26 as parallel mediating variables (See Figure 1). After conducting model analysis using AMOS 26.0, the data revealed good model fit [34] indices, suggesting an adequate fit for the model: χ^2^/df = 2.46 (value < 3 indicates a good fit); CFI = 0.93 (value > 0.90 indicates a good fit); TLI = 0.92 (value > 0.90 indicates a good fit); RMSEA = 0.05 (value < 0.06 indicates a good fit); SRMR = 0.06 (value < 0.08 indicates a good fit). These indices collectively suggest that the model provides a robust representation of the data.

**Figure 2:**
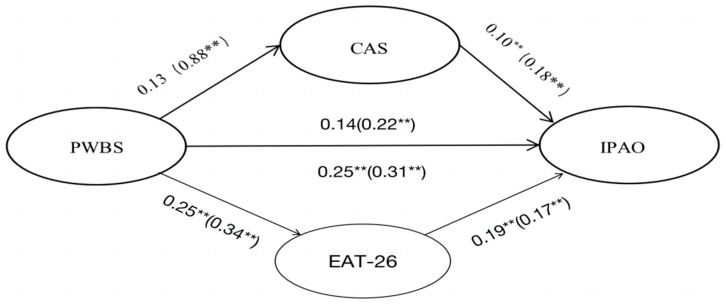
The structural of parallel mediating variable model. Values represent the significant standardized regression coefficients. The values for China are in parentheses. ** *p* < 0.01.

PWBS predicting CAS: Poland: 0.13, *p* > 0.05 (insignificant). China: 0.88, *p* < 0.01 (significant). The relationship between PWBS and CAS was significant only in China (See Figure 2). This may reflect cultural differences, where higher levels of psychological well-being in China are more strongly associated with reduced anxiety about COVID-19. In Chinese culture, where mental well-being and collective concern are deeply valued, those with better psychological well-being may be better equipped to manage health-related anxieties. In contrast, in Poland, where individualism and self-reliance are emphasized, the effect of well-being on COVID-19 anxiety may be less pronounced.

CAS predicting IPAO: Poland: 0.10, *p* < 0.01 (significant). China: 0.18, *p* < 0.01 (significant). Both Poland and China showed significant positive relationships between CAS and IPAO. This suggests that higher anxiety levels were associated with increased focus on physical activity objectives, possibly as a coping mechanism in both cultural contexts, albeit to different extents.

PWBS predicting IPAO: Poland: 0.14, *p* > 0.05 (insignificant). China: 0.22, *p* < 0.01 (significant). The effect of PWBS on IPAO was significant in China but not in Poland. This could be due to the role of well-being in shaping physical activity goals in China, where holistic health and wellness are often emphasized in both individual and social contexts. In Poland, this relationship might be weaker, reflecting a less pronounced cultural emphasis on the interconnectedness of mental and physical health.

PWBS predicting EAT-26: Poland: 0.25, *p* < 0.01 (significant). China: 0.34, *p* < 0.01 (significant). Both countries showed significant relationships between PWBS and EAT-26. This indicates that better psychological well-being is linked to healthier eating behaviors in both Polish and Chinese participants, suggesting that well-being has a universal positive influence on eating attitudes.

EAT-26 predicting IPAO: Poland: 0.19, *p* < 0.01 (significant). China: 0.17, *p* < 0.01 (significant). The relationship between EAT-26 and IPAO was significant in both countries, indicating that healthier eating attitudes are associated with more defined physical activity objectives. While the effect sizes are similar, the relationship could vary in terms of the cultural significance placed on food and physical activity, particularly in how each country views the role of physical health in overall well-being.

**Figure 3:**
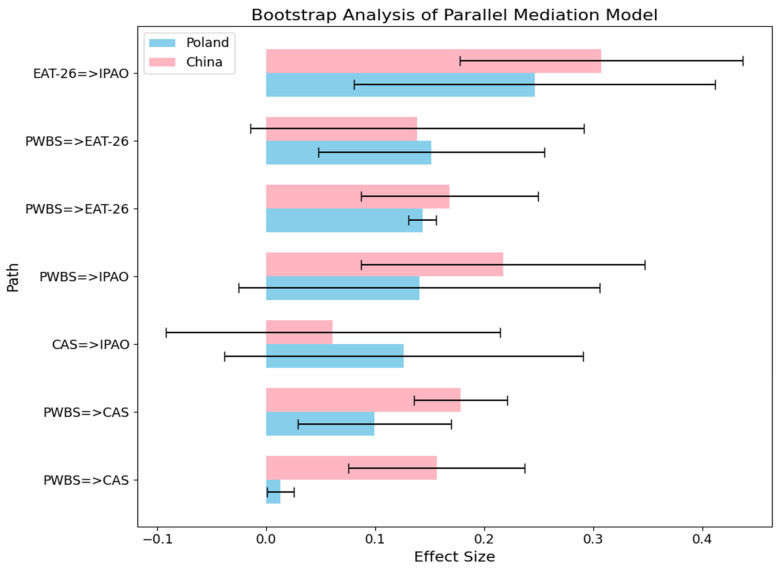
Bootstrap analysis of mediation effect size and significance test in Poland and China. *n* Poland = 644, *n* China = 690. Values represent the standardized effect size.

The data show that when CAS is used as a mediating variable, the mediating effect is insignificant in Poland, whereas in China, significant full mediating effects are observed in this study. This suggests that the effect of CAS on IPAO differs between the two countries. The reasons for these differences can be attributed to different social and cultural factors, epidemiologic control measures, demographic characteristics, and so on. These factors will be explored in detail in the discussion section. When EAT-26 was used as a mediating variable, both Polish and Chinese samples showed a significant mediating effect. Thus, that the mediating effects of COVID-19 anxiety and eating attitude on well-being and physical activity objectives differ between the two countries, was partially confirmed. Table 3 summarizes the indirect, direct and total effect values for Poland and China. Figure 3 is a visualization of the indirect, direct and total effect values for Poland and China.

**Figure 4:**
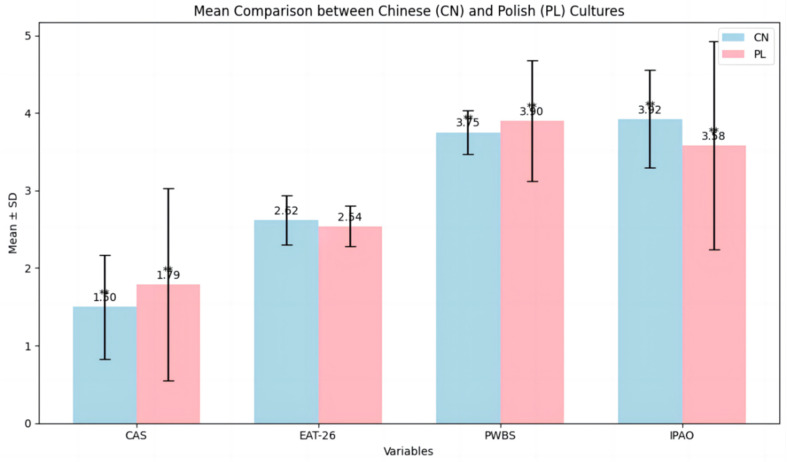
ANOVA histogram for CAS, EAT-26, PWBS, IPAO in Poland and China. *n* Poland = 644, *n* China = 690. ** *p* < 0.01.

This study utilized analysis of variance (ANOVA) to compare the means of four variables in Poland and China (See Table 4). Figure 4 is a visualization of the ANOVA. The comparison of means for PWBS produced a result of (F = 29.085, *p* = 0.00). The comparison for CAS yielded (F = 35.274, *p* = 0.00). For EAT-26, the results showed no significant differences between the countries (F = 29.058, *p* = 0.153). Similarly, the comparison for IPAO showed (F = 35.415, *p* = 0.00). These data indicate significant differences between Poland and China in all variables except EAT-26.

The results of the study indicate that the Polish sample had significantly higher levels of well-being than the Chinese sample. However, the Chinese sample exhibited a greater willingness to be motivated by physical activity compared to the Polish sample and also reported significantly lower levels of anxiety related to COVID-19. There were no significant differences in eating attitudes (EAT-26 scores) between Poland and China. In conclusion, these results suggest that significant cultural and social differences influence well-being, physical activity motivation, and COVID-19 anxiety between the two countries, but no such differences were found in eating attitudes.

### 3.4. Relationships Among PWBS, CAS, EAT-26 and IPAO Scores

**Figure 5:**
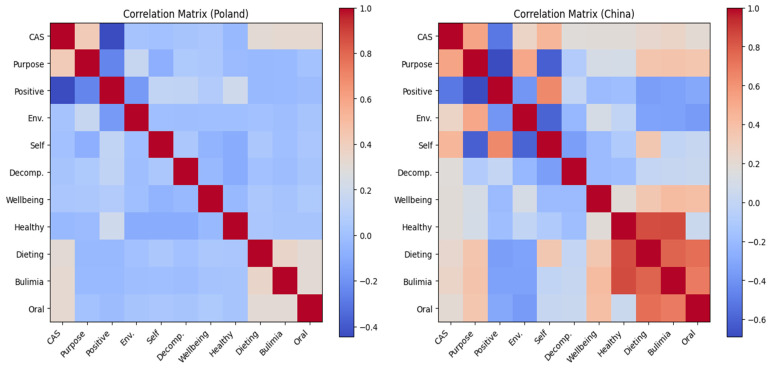
Poland and China correlation coefficient score. The color of each square represents the correlation between the two variables, with darker colors indicating negative correlations and lighter colors indicating positive correlations.

In the Polish sample, correlation analysis was employed to investigate the relationships among CAS, EAT-26 and the seven dimensions of self-acceptance, environmental mastery, positive relations, autonomy, Stress Reduction, well-being, and health. Pearson correlation coefficients were used to quantify the strength of these relationships. Specifically, the analysis revealed significant correlations between CAS and purpose, positive relations, and well-being (See Table 5). Figure 5 is a visualization of the correlation analysis of CAS, PWBS, EAT-26 and IPAO.

The correlation coefficient between CAS and purpose was 0.144, demonstrating significance at the 0.01 level, indicating a significant positive correlation. CAS and positive relations exhibited a correlation coefficient of −0.109, significant at the 0.01 level, indicating a significant negative correlation. This negative correlation suggests that as CAS scores increase, the quality of positive relationships may decrease. This could be due to a variety of factors, such as individuals with higher CAS experiencing challenges in social interactions, which may hinder the development of positive relations.

In contrast, other dimensions, such as purpose and well-being, showed positive correlations with CAS, indicating that individuals with higher CAS scores generally experienced greater sense of purpose and well-being.

CAS did not show significant correlations (*p* > 0.05) with environmental mastery, self-acceptance, Stress Reduction, well-being, and health, indicating no significant relationships between CAS and these five dimensions.

In the correlation analysis of eating attitudes, a significant positive correlation was found with health in the physical activity objectives. A significant positive correlation was also observed between CAS and all variables. Moreover, dieting, bulimia, and oral control in eating attitudes had significant positive correlations with all dimensions in PWBS. As in the Polish sample, significant positive correlations existed only with health in physical activity objectives. Therefore, significant correlations among the four scales were confirmed.

## 4. Discussion

This study aimed to investigate the factors influencing physical activity objectives across different countries during the COVID-19 pandemic, as well as the mediating role of COVID-19 anxiety and eating attitudes on well-being and physical activity objectives. The analysis also explored how cultural differences shape the relationships among well-being, COVID-19 anxiety, eating attitudes, and physical activity objectives.

### 4.1. Hypothesis 1—Mediating Role of COVID-19 Anxiety

The study revealed significant differences in the mediating role of COVID-19 anxiety between Poland and China. In Poland, COVID-19 anxiety did not significantly mediate the relationship between psychological well-being and physical activity objectives. This absence of mediation can be attributed to both cultural differences and individual coping mechanisms [35]. For example, Polish society may have adapted more quickly to pandemic restrictions [36], leading to less emotional distress. Conversely, the more stringent and prolonged lockdown measures in China, along with a collectivist culture emphasizing community well-being, likely contributed to higher levels of anxiety [37].

Cultural dimensions, particularly the Uncertainty Avoidance Index (UAI), help explain these findings [38]. Poland’s higher UAI indicates greater sensitivity to uncertainty, which likely contributed to increased anxiety levels. However, the impact of this anxiety was likely mitigated by Poland’s strong social support networks and government interventions. In contrast, China’s strict lockdown measures and strong societal expectations for conformity led to a complete mediation effect of COVID-19 anxiety [39], reflecting the profound emotional and psychological effects of the pandemic.

### 4.2. Hypothesis 2—Differences in Variables

A key finding of this study was the significant difference in psychological well-being and physical activity motivation between Polish and Chinese participants. In Poland, individuals displayed higher levels of psychological well-being and were more motivated to engage in physical activity compared to their Chinese counterparts. This difference is influenced by cultural values, such as individualism in Poland, which fosters personal autonomy and self-care [40]. In contrast, China’s collectivist culture, which prioritizes community over individual needs, may have reduced the focus on personal physical activity during the pandemic, especially under strict lockdown conditions.

Economic factors also played a role in these differences [41]. Poland’s higher national income and standard of living likely contributed to greater access to resources and better mental health outcomes, promoting higher motivation for physical activity. In China, economic disparities and the pressures of a rapidly changing society may have impeded individuals’ ability to sustain high levels of motivation.

Regarding eating attitudes, there were no significant differences between China and Poland. However, it is important to address the complexity of eating behaviors across cultures. While eating disorders are a global issue, the cultural context plays a significant role in shaping eating attitudes [42]. In Western societies like Poland, eating disorders are often linked to individualism, body image pressures, and personal autonomy. In contrast, in China, eating attitudes may be more closely tied to societal expectations and collective identity. These cultural factors influence how eating attitudes mediate physical activity objectives, suggesting that while the behaviors may appear similar, the underlying societal and psychological factors differ across cultures.

### 4.3. Hypothesis 3—Correlations

The study also examined the relationships between COVID-19 anxiety, psychological well-being (self-acceptance, environmental mastery, positive relations, autonomy), physical activity goals (well-being, health, stress reduction), and eating attitudes (dieting, bulimia, food preoccupation, oral control). Correlation analysis revealed notable differences between the Polish and Chinese samples.

For Poland, COVID-19 anxiety was significantly correlated with autonomy, positive relations, and well-being. Anxiety was negatively related to positive relations, indicating that lockdowns inhibited social interactions and lowered happiness. A positive correlation with autonomy suggested that higher self-awareness and future planning were linked to greater anxiety due to concerns about disrupted life goals. Anxiety was also positively correlated with well-being objectives in physical activity and all eating attitude dimensions, supporting findings that increased anxiety exacerbates eating disorder symptoms.

For China, COVID-19 anxiety was correlated with all variables, with a stronger association than in Poland. Anxiety was negatively correlated with self-acceptance and environmental mastery, supporting prior research that these factors buffer negative emotions. Anxiety was also positively correlated with health and stress reduction goals in physical activity, reflecting a desire to improve health and reduce stress in the face of pandemic-related fears. Eating attitudes in China were positively correlated with autonomy, environmental mastery, positive relations, and self-acceptance, suggesting that psychological well-being motivates engagement in physical activities and healthy behaviors. However, eating attitudes did not directly impact physical activity goals related to health and stress reduction.

These findings underscore the influence of cultural differences on the relationships between COVID-19 anxiety, well-being, and eating attitudes. In Poland, anxiety appears to influence autonomy and the pursuit of personal well-being through physical activity, while in China, anxiety is more strongly associated with health-related goals and eating behaviors. This suggests that cultural values and societal structures significantly shape individuals’ responses to anxiety and their subsequent engagement in physical and eating behaviors [43]. By focusing on the role of cultural differences, particularly in relation to eating attitudes, emphasizes how these cultural factors influence dietary behaviors and health outcomes across countries.

The present study contributes to the mechanisms linking anxiety, eating attitude, well-being, and objectives for physical activity in COVID-19. However, the following limitations and unanswered questions remain. The characteristics of the Polish and Chinese samples are subtly different. While the Chinese sample consisted mostly of current university students, the Polish sample was broad, covering a wide range of age groups. A cautious inference should therefore be made that the differences in the age groups of the samples may have contributed to some extent to the differences in the results between Poland and China. In a more general way, the present study is only a preliminary investigation of the reasons for the differences in correlations between COVID-19 anxiety and other variables in Poland and China. It will continue to analyze the multiple influencing factors behind this in future studies.

## 5. Conclusions

This research explored the mechanisms linking well-being, COVID-19 anxiety, and physical activity objectives, focusing on the mediating roles of COVID-19 anxiety and eating attitudes. The results showed that, in China, COVID-19 anxiety fully mediated the relationship between well-being and physical activity objectives. However, in Poland, COVID-19 anxiety did not mediate this relationship. The study concluded that COVID-19 anxiety influenced well-being and physical activity objectives both directly and indirectly, with cultural differences playing a significant role. Eating problems are globally prevalent and unaffected by cultural differences. The study highlights the significance of COVID-19 anxiety, eating disorders, and physical activity objectives, providing insights into their interrelationships across different cultural contexts. This study contributes to the fields of psychology and physical activity by addressing a significant research gap in cross-cultural studies, particularly between Poland and China.

## Figures and Tables

**Table 1:** Descriptive analysis of all variables.

Variable	Mean	SD	Min.	Max.	Effect Size (d)
Age	32.69 (24.9)	11.52 (8.51)	18 (18)	72 (64)	0.82
PWBS					
Self-Acceptance	4.03 (4.18)	0.97 (1.22)	1 (1)	6 (6)	0.27
Environmental mastery	3.45 (3.56)	1.13 (1.34)	1 (1)	6 (6)	0.29
Positive relations	4.75 (4.68)	0.84 (1.07)	1 (1)	6 (6)	0.33
Purpose in life	3.44 (3.69)	1.05 (1.31)	1 (1)	6 (6)	0.19
Personal growth	3.82 (3.99)	1.11 (1.32)	1 (1)	6 (6)	0.13
Autonomy	3.00 (3.31)	1.11 (1.42)	1 (1)	6 (6)	0.68
CAS					
I felt dizzy, lightheaded, or faint, when I read or listened to news about COVID-19.	1.59 (1.88)	0.98 (1.39)	1 (1)	5 (5)	0.84
I had trouble falling or staying asleep because I was thinking about COVID-19.	1.64 (1.95)	1.06 (1.43)	1 (1)	5 (5)	0.91
I felt paralyzed or frozen when I thought about or was exposed to information about COVID-19.	1.66 (1.81)	0.94 (1.32)	1 (1)	5 (5)	0.77
I lost interest in eating when I thought about or was exposed to information about COVID-19.	1.36 (1.72)	0.78 (1.32)	1 (1)	5 (5)	0.93
I felt nauseous or had stomach problems when I thought about or was exposed to information about COVID-19.	1.23 (1.57)	0.61 (1.28)	1 (1)	5 (5)	0.76
EAT-26					
Dieting	2.45 (2.51)	0.52 (0.43)	1 (1)	3 (3)	0.81
Bulimia	2.50 (2.55)	0.46 (0.63)	1 (1)	3 (3)	0.66
Oral control	2.68 (2.82)	0.57 (0.51)	1 (1)	3 (3)	0.57
IPAO					
Health	4.22 (3.78)	0.95 (1.53)	1 (1)	5 (5)	0.63
Well-being	3.73 (3.34)	1.09 (1.54)	1 (1)	5 (5)	0.56
Stress Reduction	3.82 (3.68)	1.12 (1.53)	1 (1)	5 (5)	0.38

Note. *n* Poland = 644, *n* China = 690. Values for China are in parentheses. Cohen’s d, criteria: small = 0.20; medium = 0.50, and large = 0.80.

**Table 2:** Factor loadings of all indicators.

Variable	Factor Loadings
PWBS	
Acceptance	0.51 (0.58)
Environmental mastery	0.57 (0.60)
Positive relations	0.66 (0.63)
*Purpose in life*	0.36 (0.59)
*Personal growth*	0.37 (0.60)
Autonomy	0.67 (0.62)
CAS	
Indicator 1: I felt dizzy, lightheaded, or faint, when I read or listened to news about COVID-19	0.74 (0.89)
Indicator 2: I had trouble falling or staying asleep because I was thinking about COVID-19	0.73 (0.88)
Indicator 3: I felt paralyzed or frozen when I thought about or was exposed to information about COVID-19	0.76 (0.91)
Indicator 4: I lost interest in eating when I thought about or was exposed to information about COVID-19	0.84 (0.94)
Indicator 5: I felt nauseous or had stomach problems when I thought about or was exposed to information about COVID-19	0.80 (0.93)
EAT-26	
Dieting	0.73 (0.68)
Bulimia	0.66 (0.75)
Oral control	0.57 (0.64)
IPAO	
Health	0.59 (0.88)
Well-being	0.68 (0.90)
Stress Reduction	0.64 (0.79)

Note. *n* Poland = 644, *n* China = 690. Values for China are in parentheses. Indicators in italics have been removed due to factor loadings below 0.5.

**Table 3:** Bootstrap analysis of the mediation effect size and significance test of CAS and EAT-26 in PWBS and IPAO.

Path		Standardized Effect Size (Effect)	95% CI		*p*
			LL	UL	
PWBS⇒CAS⇒IPAO	Indirect effect	0.013 (0.156 **)	−0.005 (0.005)	0.020 (0.167)	0.033 (0.000)
PWBS⇒CAS	X⇒M1	0.134 (0.877 **)	−0.045 (0.775)	0.313 (0.979)	0.142 (0.000)
CAS⇒IPAO	M1⇒Y	0.099 ** (0.178 **)	0.029 (0.083)	0.169 (0.272)	0.006 (0.000)
PWBS⇒IPAO	Direct effect	0.126 (0.061)	−0.038 (−0.092)	0.291 (0.214)	0.134 (0.436)
PWBS⇒IPAO	Total effect	0.140 (0.217 **)	−0.026 (0.087)	0.305 (0.347)	0.099 (0.001)
PWBS⇒EAT-26⇒IPAO	Indirect effect	0.143 ** (0.168 **)	0.004 (0.005)	0.032 (0.167)	0.003 (0.000)
PWBS⇒EAT-26	X⇒M2	0.255 ** (0.341 **)	0.035 (0.775)	0.352 (0.979)	0.001 (0.001)
EAY-26⇒IPAO	M2⇒Y	0.189 ** (0.171 **)	0.029 (0.083)	0.169 (0.272)	0.001 (0.001)
PWBS⇒IPAO	Direct effect	0.151 ** (0.138 **)	0.029 (0.092)	0.371 (0.315)	0.002 (0.001)
PWBS⇒IPAO	Total effect	0.246 ** (0.307 **)	0.016 (0.037)	0.205 (0.345)	0.001 (0.001)

Note: CI confidence interval, LL lower limit, UL upper limit. X independent variable, M mediating variable, Y dependent variable. The values for China are in parentheses. ** *p* < 0.01. Bolded values represent significant differences.

**Table 4:** ANOVA Analysis of CAS, EAT-26, PWBS, IPAO.

	Culture (Mean ± SD)		F Value	*p* Value
	China	Poland		
CAS	1.50 ± 0.67	1.79 ± 1.24	35.274	0.000 **
EAT-26	2.62 ± 0.32	2.54 ± 0.26	31.335	0.153
PWBS	3.75 ± 0.28	3.90 ± 0.78	29.058	0.000 **
IPAO	3.92 ± 0.63	3.58 ± 1.34	35.415	0.000 **

Note: ** *p* < 0.01.

**Table 5:** Pearson correlation between CAS, EAT-26, PWBS, and IPAO scores.

	1	2	3	4	5	6	7	8	9	10	11
CAS (1)	1										
Autonomy (2)	0.413 ** (0.526 **)	1									
Positive relations (3)	−0.445 ** (0.512 **)	−0.250 ** (0.689 **)	1								
Environment mastery (4)	0.018 (0.259 **)	0.161 ** (0.344 **)	−0.171 ** (0.391 **)	1							
Self-acceptance (5)	0.012 (0.444 **)	−0.079 * (0.612 **)	0.144 ** (0.643 **)	−0.001 (0.592 **)	1						
Stress Reduction (6)	0.026 (0.170 **)	0.054 (0.085 *)	0.136 ** (−0.006)	0.046 (0.216 **)	−0.013 (0.109 **)	1					
Wellbeing (7)	0.037 (0.179 **)	0.042 (0.107 **)	0.069 (−0.004)	−0.058 (0.197 **)	−0.036 (0.109 **)	0.502 ** (0.914 **)	1				
Healthy (8)	−0.036 (0.177 **)	−0.027 (0.098 *)	0.201 ** (−0.001)	−0.097 * (0.179 **)	−0.034 (0.103 **)	0.466 ** (0.846 **)	0.649 ** (0.849 **)	1			
Dieting (9)	0.308 ** (0.228 **)	−0.034 (0.357 **)	−0.035 (0.329 **)	0.009 (0.346 **)	0.044 (0.407 **)	0.037 (0.065)	0.019 (0.070)	0.316 ** (0.466 **)	1		
Bulimia and Food preoccupation (10)	0.320 ** (0.254 **)	−0.031 (0.370 **)	−0.031 (0.334 **)	−0.005 (0.337 **)	0.025 (0.407 **)	0.018 (0.049)	−0.014 (0.055)	0.351 ** (0.356 **)	0.770 ** (0.928 **)	1	
Oral control (11)	0.326 ** (0.198 **)	0.016 (0.349 **)	−0.020 (0.303 **)	0.026 (0.360 **)	0.038 (0.394 **)	0.053 (0.049)	0.026 (0.066)	0.306 ** (0.363 **)	0.742 ** (0.941 **)	0.690 ** (0.913 **)	1

Note: *n* Poland = 644, *n* China = 690. Values for China are in parentheses. * *p* < 0.05, ** *p* < 0.01.

## Data Availability

The datasets generated during and analyzed during the current study are available from [44].
